# Risk Factors for Hantavirus Infection in Germany, 2005

**DOI:** 10.3201/eid1309.070552

**Published:** 2007-09

**Authors:** Muna Abu Sin, Klaus Stark, Ulrich van Treeck, Helga Dieckmann, Helmut Uphoff, Wolfgang Hautmann, Bernhard Bornhofen, Evelin Jensen, Günter Pfaff, Judith Koch

**Affiliations:** * Robert Koch Institute, Berlin, Germany; † Institute of Public Health, Muenster, North-Rhine Westphalia, Germany; ‡ Regional Health Authority, Hanover, Lower Saxony, Germany; § Government Health Service Institute, Dillenburg, Hesse, Germany; ¶ Bavarian Health and Food Safety Authority, Munich, Bavaria, Germany; # Institute for Hygiene and Infection Control, Landau, Rhineland-Palatinate, Germany; ** Thuringian State Authority for Food Safety and Consumer Protection, Erfurt, Thuringia, Germany; †† State Health Office, Stuttgart, Baden-Wuerttemberg, Germany

**Keywords:** Hantavirus infection, Puumala virus, hemorrhagic fever with renal syndrome, outbreak, case–control study, risk factor, dispatch

## Abstract

In 2005, a marked increase in hantavirus infections was observed in Germany. Large cities and areas where hantaviruses were not known to be endemic were affected. A case–control study identified the following independent risk factors for infection: occupational exposure for construction workers, living <100 m from forested areas, and exposure to mice.

Hantaviruses (family *Bunyaviridae*) are rodentborne pathogens found worldwide. They have caused recurrent epidemics in several countries (*1*–*3*). Hantaviruses circulating in North and South America can cause a fatal cardiopulmonary syndrome; hantavirus infections in Europe and Asia can result in a hemorrhagic fever with renal syndrome (HFRS) of varying severity. In Germany, the predominant serotype is Puumala; its main reservoir is bank voles (*Myodes glareolus*). A mild form of HFRS (nephropathia epidemica) usually develops in patients, but a substantial number require hospitalization and hemodialysis (*1*).

In 2001, hantavirus infection became a mandatory reportable disease in Germany. From January through May 2005, the number of reported case-patients (n = 158) almost tripled when compared with the number of patients seen in the same period in previous years. Unexpectedly, infections were also observed in larger cities and in rural regions where they were not known to have occurred previously. Thus far, risk factors for hantavirus infections have been assessed in rural settings (*3*–*5*). The unusual geographic pattern in 2005 in Germany prompted us to conduct a case–control study to investigate potentially new risk factors for human hantavirus infections.

## The Study

In Germany, all laboratory-confirmed hantavirus infections are reported to the local public health authorities and forwarded through the federal states to the Robert Koch Institute. Laboratory diagnosis is based on detection of nucleic acid, a marked rise of immunoglobulin (Ig) G antibodies in a paired sample, or detection of IgM or IgA antibodies confirmed by IgG antibodies. Local health departments identified eligible case-patients for the case–control study according to the following criteria: laboratory-confirmed hantavirus infection with clinical symptoms acquired in Germany and a reporting date between May and August 2005. Controls were selected from the population by sequential digital telephone dialing and matched individually by sex and residential area. An exclusion criterion for controls was having had a diagnosis of hantavirus infection or a disease with fever (>38.5°C) for at least 3 days, accompanied by back pain, abdominal pain, or headache in the 4 weeks preceding the interview. All participants were >18 years of age and provided informed consent.

Interviews were conducted by trained public health professionals who used a standardized questionnaire. The questionnaire covered demographic, clinical, and exposure data, e.g., type of residential area, residence distance from forested areas, handling of wood, outdoor activities, occupational exposures, contact with rodents and rodents’ droppings, and travel history. The relevant period of exposure was 4 weeks preceding disease onset for case-patients and 4 weeks preceding the interview for controls. Controls were interviewed on average within 2 weeks after case-patients.

Matched odds ratios were calculated and variables with p<0.2 were considered for the conditional logistic regression model by using EpiInfo 3.3.2 (Centers for Disease Control and Prevention, Atlanta, GA, USA) and SAS statistical package version 8 (SAS Institute, Cary, NC, USA). A forward stepwise procedure was chosen. Variables with p<0.05 remained in the model. For each step the more complex model was compared with the previous model on the basis of likelihood ratio statistics. Selected variables were examined for collinearity, and interaction was tested in the conditional logistic regression model.

**Figure 1:**
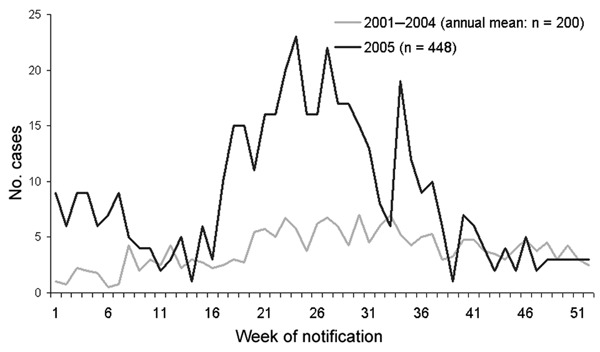
Reported hantavirus infections in 2005 compared with the annual average in 2001–2004, by week of report, Germany.

**Figure 2:**
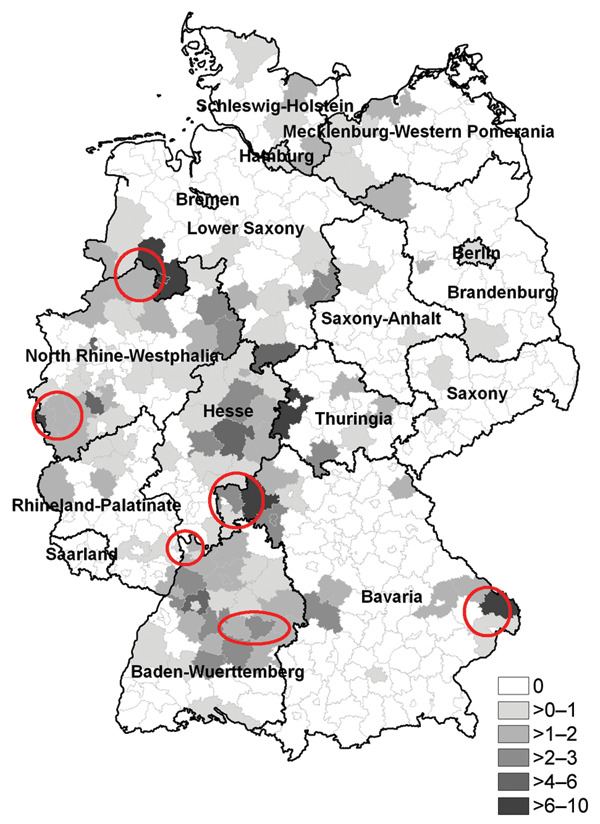
Incidences of reported hantavirus infections per 100,000 inhabitants by administrative district, Germany, 2005. Circles represent areas in which hantaviruses were known to be endemic.

In the total year 2005, 448 hantavirus case-patients were reported. Notifications increased steeply in May and persisted at a high level until August. The annual incidence (0.54/100,000 inhabitants) almost doubled compared with that in 2001–2004 (Figure 1). Particularly high incidences were observed in some cities (Osnabrück 8.5, Aachen 8.1, and Cologne 4.2) (Figure 2).

In the case–control study (May–August), 154 (71.6%) of 215 eligible case-patients participated, and 150 matched case–control pairs were analyzed. The male:female ratio was 2.1:1. The median age of case-patients was 42 years (range 19–75) and of controls, 46 years (range 20–87) (p<0.01). Of all case-patients, 40.7% lived in rural areas and 32.7% in cities with >100,000 inhabitants. Most case-patients had fever (87.7%); other major symptoms included back pain (74.8%), headache (73.9%), myalgia (73.7%), nausea (68.0%), vomiting (50.7%), blurred vision (45.3%), and abdominal pain (41.6%). Median duration of symptoms was 12 days. Of the case-patients, 73.4% were hospitalized (median duration 8 days), and 6.6% required hemodialysis. No hemorrhagic or fatal course was reported during the outbreak. Of the employed patients, 92.2% reported absence from work because of hantavirus infection (median duration 19 days).

Table 1 shows the associations of different exposure variables with the outcome in univariate analysis. Of note, 12.2% of the case-patients were forestry workers, and 11.5% were construction workers (27.5% of the male case-patients). Risk factors did not differ significantly between case-patients from urban and rural areas. Occupational exposure as a construction worker, noticing mice in the neighborhood, and living in a building <100 m from forested areas remained independent risk factors (adjusted for age) in the multivariate model (Table 2). No significant interaction was found.

**Table 1:** Univariate matched analysis for exposure variables for hantavirus infection, conditional logistic regression, Germany, 2005*

Exposure	Case-patients, no. (%)	Controls, no. (%)	Matched OR	95% CI	p value
Noticing mice	75 (50.0)	48 (32.0)	2.5	1.4–4.5	<0.01
In forested areas	28 (18.7)	4 (2.7)	13.0	3.3–113.0	<0.01
Noticing mice droppings	43 (28.7)	21 (14.0)	2.5	1.3–5.0	<0.01
Living <100 m from forested areas	65 (43.3)	39 (26.0)	2.3	1.3–4.1	<0.01
Being a forestry worker	18 (12.2)	8 (5.4)	2.7	1.0–8.3	0.05
Being a construction worker	17 (11.5)	5 (3.4)	4.0	1.3–16.4	0.01
Entering empty rooms or buildings	26 (17.3)	10 (6.7)	2.8	1.3–6.8	0.01
Cutting or handling wood	35 (23.3)	21 (14.0)	2.0	1.0–4.2	0.05
Gardening	85 (56.7)	98 (65.8)	0.7	0.4–1.1	0.14

*OR, odds ratio; CI, confidence interval.

**Table 2:** Risk factors for hantavirus infection, conditional logistic regression model, Germany, 2005

Exposure	Odds ratio*	95% Confidence interval	p value
Working in construction	4.8	1.4–17.1	0.01
Noticing mice	3.0	1.6–6.0	<0.01
Living <100 m from forested areas	2.5	1.3–4.7	<0.01

*Adjusted for age.

## Conclusions

The 2005 hantavirus epidemic in Germany caused substantial disease and showed remarkable epidemiologic characteristics. Compared with data from previous years, a relatively early and steep increase in patient numbers was observed in May, and the high disease activity extended over several months. A substantial number of patients acquired their infection in areas where the disease was previously not known to be endemic, most notably in urban settings near forests and wooded municipal parks. The main reason for the epidemic was a strong rise in the reservoir population, which has its habitat in forested areas. In fact, in some places an upsurge in the bank vole population had already occurred in 2004 because of the intense beech mast (F. Krüger, pers. commun.). Most likely, hantavirus-infected bank voles were also increasingly present in forested parts of inner city areas. In Cologne, environmental investigations detected Puumala virus in 66% of trapped bank voles (*6*). It is unclear, however, whether the virus has been newly introduced in these areas or had been present previously but only at very low levels or in small ecologic niches constituting only negligible risks for humans. A systematic monitoring system for rodents (population density, hantavirus prevalence) could be used to predict an increased human risk and facilitate recommendations for persons in at-risk areas.

Living close to forested areas was a major risk factor independent of a residence in more rural or urban areas. A substantial number of case-patients probably acquired infection in an area close to where they lived. Leisure activities in forested areas did not significantly increase the risk for hantavirus infection, as has been reported in other studies (*4*). If areas close to human residences are increasingly contaminated with virus-containing rodent excreta, the inhabitants are more likely exposed by common activities such as cleaning up around houses or sheds. Persons living close to areas with hantavirus-infected rodent populations should be informed about the potential exposure risks and follow recommendations for prevention and control (*7*). In residential areas, rodent control measures should be maintained at a high level.

Almost 30% of male case-patients were construction or forestry workers. Probably because of such occupational differences, but also because of recreational exposure patterns, men were predominantly affected in this epidemic, as has been described in other countries (*1*,*5*,*8*). Most case-patients who were construction workers mentioned having worked on restoring old buildings during the likely incubation period. Building sites near forested areas (and particularly older buildings in need of restoration) are likely infested by bank voles and pose considerable hazards to humans who work there. Several studies have shown that forestry workers, farmers, or soldiers in maneuvers are at increased risk (*4*,*5*,*9*,*10*).

In 2005, a similar marked increase of hantavirus infection was observed in Belgium and France, but case-patients living in more densely populated urban areas were reported only from Germany (*11*). To better understand the dynamics of the reservoir population as well as the epidemiologic characteristics and risk factors among humans, a concerted approach to monitoring of the reservoir and to surveillance and investigation of human cases are warranted in neighboring countries.
